# Electrospun Poly(lactide-*co*-glycolide-*co*-3(*S*)-methyl-morpholine-2,5-dione) Nanofibrous Scaffolds for Tissue Engineering

**DOI:** 10.3390/polym8020013

**Published:** 2016-01-29

**Authors:** Yakai Feng, Wei Lu, Xiangkui Ren, Wen Liu, Mengyang Guo, Ihsan Ullah, Wencheng Zhang

**Affiliations:** 1 School of Chemical Engineering and Technology, Collaborative Innovation Center of Chemical Science and Chemical Engineering, Tianjin University, Tianjin 300072, China; yakaifeng@tju.edu.cn (Y.F.); care1990@tju.edu.cn (Wei L.); 18202520278@163.com (Wen L.); 15002257297@163.com (M.G.); ihsan.chem@tju.edu.cn (I.U.); 2 Tianjin University-Helmholtz-Zentrum Geesthacht, Joint Laboratory for Biomaterials and Regenerative Medicine, Tianjin 300072, China; 3 Key Laboratory of Systems Bioengineering of Ministry of Education, Tianjin University, Tianjin 300072, China; 4 Department of Physiology and Pathophysiology, Logistics University of Chinese People’s Armed Police Force, Tianjin 300162, China

**Keywords:** electrospinning, nanofibrous scaffolds, vascular tissue engineering, copolymers

## Abstract

Biomimetic scaffolds have been investigated in vascular tissue engineering for many years. Excellent biodegradable materials are desired as temporary scaffolds to support cell growth and disappear gradually with the progress of guided tissue regeneration. In the present paper, a series of biodegradable copolymers were synthesized and used to prepared micro/nanofibrous scaffolds for vascular tissue engineering. Poly(lactide-*co*-glycolide-*co*-3(*S*)-methyl-morpholine-2,5-dione) [P(LA-*co*-GA-*co*-MMD)] copolymers with different l-lactide (LA), glycolide (GA), and 3(*S*)-methyl-2,5-morpholinedione (MMD) contents were synthesized using stannous octoate as a catalyst. Moreover, the P(LA-*co*-GA-*co*-MMD) nanofibrous scaffolds were prepared by electrospinning technology. The morphology of scaffolds was analyzed by scanning electron microscopy (SEM), and the results showed that the fibers are smooth, regular, and randomly oriented with diameters of 700 ± 100 nm. The weight loss of scaffolds increased significantly with the increasing content of MMD, indicating good biodegradable property of the scaffolds. In addition, the cytocompatibility of electrospun nanofibrous scaffolds was tested by human umbilical vein endothelial cells. It is demonstrated that the cells could attach and proliferate well on P(LA-*co*-GA-*co*-MMD) scaffolds and, consequently, form a cell monolayer fully covering on the scaffold surface. Furthermore, the P(LA-*co*-GA-*co*-MMD) scaffolds benefit to excellent cell infiltration after subcutaneous implantation. These results indicated that the P(LA-*co*-GA-*co*-MMD) nanofibrous scaffolds could be potential candidates for vascular tissue engineering.

## 1. Introduction

Cardiovascular diseases are one of the most frequent causes of death all over the world [1]. In the last three decades the demand for artificial blood vessels has increased due to a higher incidence of cardiovascular diseases [2]. Tissue engineering is emerging as a potential solution for tissue and organ transplantations [3]. However, the small-diameter, synthetic vascular grafts are encountered in several problems such as thrombosis, intimal hyperplasia, and lower long-term patency rates in clinical application [1,4]. The ideal vascular tissue engineering scaffolds should be designed to mimic the native extracellular matrix (ECM) in order to regulate cellular behavior [5]. Native ECM is an interconnected 3D porous structure consisting of proteins and carbohydrate biopolymers, which provides suitable environment for cells to adhere, grow, and proliferate [6]. Native ECM structure can be mimicked through many ways, such as drawing [7], template synthesis [8], temperature-induced phase separation [9], molecular self-assembly [10], and electrospinning [11,12,13,14,15]. Among these strategies, electrospinning technology is regarded as an efficient preparation method because it can produce nanofibrous scaffolds with interconnected pores, high porosity, and large surface area to mimic native ECM [16,17,18,19,20,21,22].

In addition, materials also play an important role in most vascular tissue engineering strategies. The ideal materials for vascular tissue engineering should have excellent biocompatibility, good mechanical properties, and appropriate degradation behavior [23]. Biodegradable polymers, such as poly(l-lactic acid) (PLLA), poly(glycolic acid) (PGA) and poly(lactic-*co*-glycolic acid) (PLGA), have drawn a lot of attention in research because of their well-characterized biodegradable properties [24,25,26,27,28]. These materials have been successfully used in bone tissue, vessel, and patches for wounds [29,30,31]. Among these biodegradable polymers, PLGA is one of the most widely used classes of polymers for vascular tissue engineering, which has been already approved by the Food and Drug Administration (FDA) as a constituent of many biomaterial-based devices [28].

Currently, polydepsipeptides have been widely accepted as a valuable class of synthetic biodegradable polymers because the strong intermolecular hydrogen-bond interaction between the amide groups can achieve a higher reactivity in ring-opening polymerization (ROP), and the presence of easily hydrolysable ester linkages and amide-bonds can ensure high degradability [32,33,34,35]. In 1985, Helder *et al.* synthesized polydepsipeptides by ROP of morpholine-2,5-dione derivatives for the first time [36]. Since then, morpholine-2,5-dione derivatives, which have an ester group and an amide group in one six-membered ring, have been used to synthesize various biodegradable copolymers [37,38,39].

In particular, 3(*S*)-methyl-morpholine-2,5-dione (MMD), which possesses a better performance of polymerization than other morpholine-2,5-dione derivatives with large substitutes, has been a promising derivative to develop biodegradable drug delivery systems [35]. Meanwhile, the copolymers synthesized from MMD shows different crystallization degrees owing to the optical activity of the monomer [35,40,41]. Moreover, the degradation products of MMD including l-amino acids can stimulate the proliferation of endothelial cells via a signal pathway and be properly metabolized by living tissues [42,43,44,45]. Based on the copolymers of MMD with other cyclic monomers, our group have developed some gene delivery systems for promoting the transfection and migration of endothelial cells [42,46,47,48,49]. However, to the best of our knowledge, the study on electrospun nanofibrous scaffolds from the copolymers of MMD with other cyclic monomers for vascular tissue engineering has not been reported previously.

In this article, poly(lactide-*co*-glycolide-*co*-3(*S*)-methyl-morpholine-2,5-dione) P(LA-*co*-GA-*co*-MMD) copolymers were synthesized by ROP of l-lactide (LA), glycolide (GA) and MMD using stannous octoate (Sn(Oct)_2_) as a catalyst. Their structural characteristics and molecular weights were identified or determined by ^1^H NMR, FT-IR, and gel permeation chromatography (GPC) analysis. Moreover, electrospinning was employed to prepare the P(LA-*co*-GA-*co*-MMD) nanofibrous scaffolds for potential use in vascular tissue engineering. The mechanical properties of electrospun P(LA-*co*-GA-*co*-MMD) scaffolds were investigated under dry and wet conditions. The degradation behavior of the scaffolds were investigated in phosphate buffered saline (PBS) solution at 37 °C for 28 days. The proliferation of human umbilical vein endothelial cells (HUVECs) on scaffolds were studied by SEM and thiazolyl blue assay (MTT). The cell infiltration of P(LA-*co*-GA-*co*-MMD) scaffolds were investigated by subcutaneous implantation.

## 2. Experimental Section

### 2.1. Materials

Sn(Oct)_2_ was purchased from Sigma-Aldrich (Beijing, China). GA and LA were obtained from Foryou Medical Device Co., Ltd. (Huizhou, China). MMD monomer was prepared using our previously reported method [50]. All the other chemicals were purchased from Tianjin Jiangtian Chemical Technology Co., Tianjin, Ltd, China. These chemicals were of analytical grade and used without further purification.

### 2.2. Synthesis of Copolymer PLGA and P(LA-co-GA-co-MMD)

#### 2.2.1. Synthesis of Copolymer PLGA

PLGA (LA/GA = 75/25, *w*/*w*) was synthesized by ROP of LA and GA using Sn(Oct)_2_ as a catalyst.

#### 2.2.2. Synthesis of P(LA-*co*-GA-*co*-MMD) Copolymers

LA, GA, MMD and Sn(Oct)_2_ toluene solution were added into a Schlenk and submitted to vacuum/nitrogen cycles. The Schlenk was finally sealed under dry nitrogen and was immersed into an oil bath previously heated to 120 °C for 24 h. The copolymer was dissolved in chloroform and precipitated in *n*-hexane, repeated this process for three times, and dried at room temperature for 24 h under vacuum. Finally, the copolymers named as P(LA-*co*-GA-*co*-MMD)_1_, P(LA-*co*-GA-*co*-MMD)_2_ and P(LA-*co*-GA-*co*-MMD)_3_ were obtained, where the subscript 1, 2, and 3 stand for different LA/GA/MMD contents in copolymers as described in Table 1.

**Table 1:** The synthesis yields, molecular weights, and contents of LA, GA, and MMD in copolymers.

Sample ID	Weight Content ^a^/%			Yield wt %	*M*_n_^b^/10^4^	*M*_w_^b^/10^4^	PDI ^b^
	LA	GA	MMD				
PLGA	70.1	29.9	0	80.3	5.19	11.89	2.29
P(LA-*co*-GA-*co*-MMD)_1_	70.3	24.2	5.5	82.1	5.74	13.35	2.33
P(LA-*co*-GA-*co*-MMD)_2_	66.1	20.9	13.0	78.4	4.67	10.88	2.33
P(LA-*co*-GA-*co*-MMD)_3_	60.9	21.7	17.4	76.8	4.08	9.44	2.32

^a^ Estimated by ^1^H NMR; ^b^ Determined by GPC; PLGA, poly(lactic-*co*-glycolic acid); LA, l-lactide; GA, glycolide; MMD, 3(*S*)-methyl-2,5-morpholinedione; PDI, polydispersity index.

### 2.3. Preparation of Spinning Solution

PLGA, P(LA-*co*-GA-*co*-MMD)_1_, P(LA-*co*-GA-*co*-MMD)_2_, and P(LA-*co*-GA-*co*-MMD)_3_ were dissolved in chloroform (CHCl_3_) and *N*,*N*-dimethylformamide (DMF) solvent in 7:3 volume ratio to prepare 20 *w*/*v* % spinning solution separately.

### 2.4. Fabrication of the Electrospun Scaffolds

The spinning solution was fed into a syringe with a blunt metal needle tip. The needle tip with the inner diameter of 0.5 mm was clamped by a high voltage power supply (Model, 0–50 kV, Tianjin Dongwen high voltage power supply company, Tianjin, China). The spinning solution was delivered by a syringe pump (749000-05, Cole-Parmer instrument company, Beijing, China) and the applied voltage, feed rate, target distance, temperature, and rotating speed of the collector were 18 kV, 0.6 mL/h, 15 cm, 25 °C, and 200 r/min, respectively. The electrospun fibers were collected on a metal plate covered with aluminum foil and placed into a vacuum oven at room temperature to evaporate residue solvents.

### 2.5. Characterization

FT-IR spectra of the copolymers were obtained using a FT-IR spectrometer (FTS-6000, Bio-Rad, Hercules, CA, USA). ^1^H NMR spectra of the synthesized copolymers were recorded with a Bruker Avance spectrometer (AV-400, Bruker, Karlsruche, Gemany) operating at 400 MHz in DMSO-d_6_.

The surface morphology of scaffolds was analyzed by field emission scanning electron microscope (S-4800, Hitachi, Tokyo, Japan) at an accelerating voltage of 10 kV, after sputter coating with gold. The fiber average diameters were calculated by measuring not less than 50 randomly selected fibers from SEM micrographs using Digimizer software (Tianyan Rongzhi, Beijing, China).

The mechanical properties of electrospun nanofibrous scaffolds were determined using a tensile testing instrument (WDW-02, Changchun, China) equipped with a 100 N load cell at a cross-head speed of 5 mm/min. The nanofibrous scaffolds were cut into rectangle with length of 20 mm, width of 5 mm and thickness measured with thickness meter, and then tested in dry and wet conditions (immersion in PBS solution for 1 h at room temperature to simulate the application condition). Five samples of each electrospun scaffold were tested to calculate an average value.

The PLGA and P(LA-*co*-GA-*co*-MMD) nanofibrous scaffolds were cut into rectangles with lengths of 20 mm, widths of 10 mm, and weighed accurately, then put them into 10 mL PBS solution (pH = 7.4) at 37 °C to test their degradation behavior. At different time intervals, the scaffolds were removed from PBS solution and washed three times with distilled water and placed in a vacuum oven for 24 h. After weighing accurately, the weight loss was calculated according to the following equation:

Weight loss (%) = (*W*_0_ − *W*_i_)/*W*_0_ × 100% (1)

where *W*_i_ is the mass of the scaffolds after degradation for i days, and *W*_0_ is the mass of the scaffolds before degradation.

### 2.6. In Vitro Cell Culture Experiment

Human umbilical vein endothelial cells (HUVECs) (Tianjin Hospital of Armed Police Forces, Tianjin, China) were cultured in Dulbecco’s modified Eagle medium (DMEM, Gibco, Waltham, MA, USA) with supplements of 10% fetal bovine serum (FBS, Gibco), 100 µg/mL penicillin, and 100 µg/mL streptomycin, in a humidified atmosphere of 5% CO_2_ at 37 °C. The adherent cells were cultured to confluence, and the medium was exchanged every two days.

### 2.7. Cell Seeding and Morphology Observation

Nanofibrous scaffolds were cut to fit the size of 96-well plate and sterilized by being soaked in 75% ethanol for 30 min, then washed thoroughly by PBS solution (pH = 7.4). Then the scaffolds were put into 96-well culture plates. HUVECs were trypsinized, counted, and then plated at a density of 1 × 10^4^ cells/well onto the surface of scaffolds. In order to avoid scaffold floating, thin glass rings fitting the inner diameter of the wells were used. Cellularized scaffolds were incubated at 37 °C in 5% CO_2_.

After 7 days of cell culture, the nanofibrous scaffolds were harvested, and washed with PBS to remove non-adherent cells and then fixed in 2.5% glutaraldehyde for 2 h at room temperature. The scaffolds were dehydrated using series of graded alcohol solutions from 50% to 100% in steps of 10% for 10 min, and then lyophilized. To evaluate cell morphology and adhesion to the scaffolds, SEM was employed at an accelerating voltage of 10 kV.

### 2.8. MTT Assay

Thiazolyl blue assay was performed at time point of one, three, and secen days to determine the relative cell viability in each well. Scaffolds were sterilized and pre-wetted as above described. HUVECs were seeded at a density of 1 × 10^4^ cells/well onto the scaffolds in 96-well culture plate. Briefly, 20 µL of MTT (5 mg/mL) was added to each well and incubated at 37 °C in 5% CO_2_ for 4 h in humidified atmosphere. At the end of assay, the purple formazan reaction product was dissolved by adding 150 µL dimethyl sulfoxide (DMSO) and vibrated slightly for 10 min, and then 100 µL solution was transferred into a new 96-well plate. The absorbance of each sample was measured using an ELISA reader at 490 nm. The relative cell viability (%) was calculated according to the following equation:

Relative cell viability = OD490′/avg(OD490′) × 100%
(2)

where OD490′ is the absorbance value of experimental well minus zero wells and avg(OD490′) is the average absorbance value of control wells minus zero wells.

### 2.9. Subcutaneous Implantation

Sixteen Sprague-Dawley rats (male, 150–200 g) were used for this study. The investigation conforms to the Guide for the Care and Use of Laboratory Animals published by the US National Institutes of Health (NIH Publication 8th Edition, 2011). The animal experimental protocol was approved by the Animal Care and Use Ethics Committee of the Logistics University of Chinese People’s Armed Police Force. The scaffolds were treated with a 70% ethanol solution overnight and then stored in sterile filtered PBS solution before implantation. These rats were randomly assigned to four groups (two specimens in each rat) to implant PLGA, P(LA-*co*-GA-*co*-MMD)_1_, P(LA-*co*-GA-*co*-MMD)_2_, and P(LA-*co*-GA-*co*-MMD)_3_ scaffolds. After seven days, the scaffolds were explanted and fixed in 10% buffered formalin solution for two days. Once the dehydration procedure was completed, each specimen was embedded in paraffin and sectioned into 5 µm slice. The sections were stained with hematoxylin and eosin (H and E) for histological analyses.

## 3. Results and Discussion

### 3.1. Synthesis of P(LA-co-GA-co-MMD) Copolymers

**Scheme 1:**
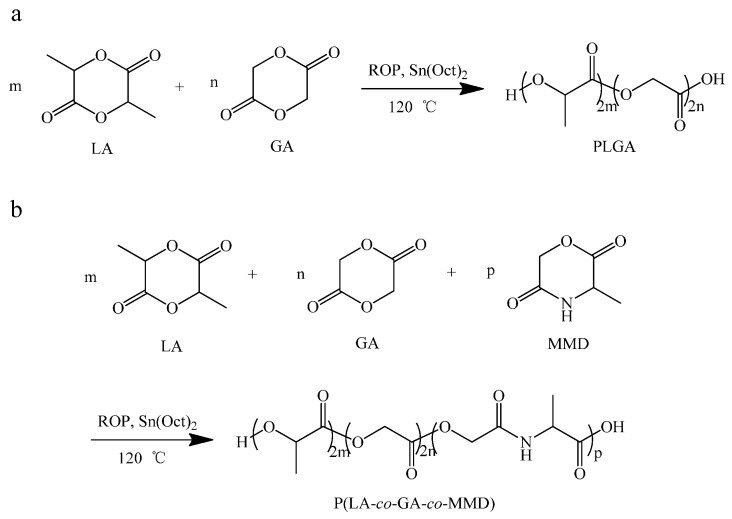
Synthesis of PLGA copolymers (**a**) and P(LA-*co*-GA-*co*-MMD) copolymers (**b**).

The synthesis of P(LA-*co*-GA-*co*-MMD) copolymers is shown in Scheme 1. The PLGA and P(LA-*co*-GA-*co*-MMD) copolymers were synthesized by ROP of LA, GA,s and MMD using Sn(Oct)_2_ as catalyst. As shown in Table 1, the contents of LA, GA and MMD in copolymers and the molecular weights of copolymers were estimated by ^1^H NMR and GPC, respectively. The yields of the copolymerization were in the range of 75%–85%. The MMD contents in P(LA-*co*-GA-*co*-MMD)_1_, P(LA-*co*-GA-*co*-MMD)_2_ and P(LA-*co*-GA-*co*-MMD)_3_ copolymers were estimated to be 5.5%, 13.0% and 17.4%, respectively. And the number-averaged molecular weights and polydispersity indexes of copolymers were in the range of 4.08× 10^4^–5.74 × 10^4^ and 2.29–2.33, respectively.

**Figure 1:**
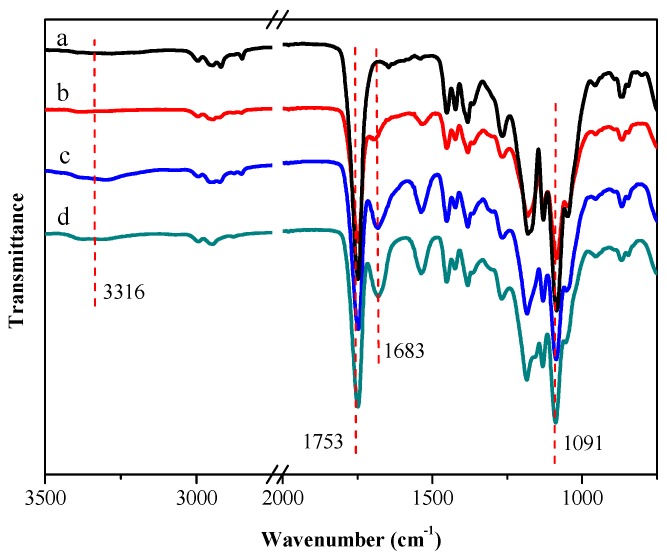
FT-IR spectra of PLGA and P(LA-*co*-GA-*co*-MMD) copolymers. (**a**) PLGA; (**b**) P(LA-*co*-GA-*co*-MMD)_1_; (**c**) P(LA-*co*-GA-*co*-MMD)_2_; and (**d**) P(LA-*co*-GA-*co*-MMD)_3_.

As shown in Figure 1, FT-IR spectra of P(LA-*co*-GA-*co*-MMD) copolymers presented a strong signal at 1753 cm^−1^ which indicated the carbonyl group (–C=O) in the copolymers. The stretching frequency at 1091 cm^−1^ indicated the ester group (–O–C=O), and the absorption bands at 1683 cm^−1^ as well as the broad absorption band from 3200 to 3400 cm^−1^ assigned to the stretching of secondary amide (–NH–) group. It was found that the characteristic peaks of the secondary amide (–NH–) was obviously displayed in the copolymers and the relative adsorption intensity of these peaks became stronger along with the increasing MMD content in P(LA-*co*-GA-*co*-MMD) copolymers. These results suggested that P(LA-*co*-GA-*co*-MMD) copolymers were synthetized successfully.

**Figure 2:**
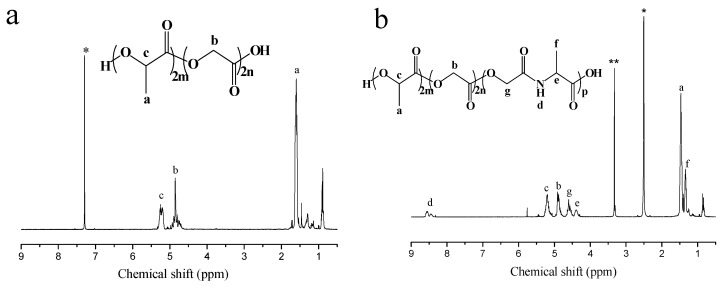
^1^H NMR spectra of PLGA and P(LA-*co*-GA-*co*-MMD)_3_ copolymers. (**a**) ^1^H NMR spectra of PLGA copolymer in CDCl_3_; (**b**) ^1^H NMR spectra of P(LA-*co*-GA-*co*-MMD) copolymer in DMSO-d_6_, * Solvent peak; ** H_2_O peaks.

The ^1^H NMR spectra of PLGA and P(LA-*co*-GA-*co*-MMD)_3_ copolymers are shown in Figure 2. The presence of methine (–CH) and methyl (–CH_3_) protons in LA was observed at around 5.21 ppm (c) and 1.50 ppm (a), respectively. The evidence for methane (–CH_2_) in GA occurred at around 4.85 ppm (b). As shown in Figure 2b, the methine (–CH), methane (–CH_2_) and methyl (–CH_3_) protons in MMD were observed at around 4.40 ppm (e), 4.60 ppm (g) and 1.35 ppm (f), respectively. The evidence for secondary amide (–NH–) in MMD occurred at around 8.53 ppm (d). These results suggested that P(LA-*co*-GA-*co*-MMD) copolymers were synthetized successfully.

### 3.2. Morphology of PLGA and P(LA-co-GA-co-MMD) Scaffolds

**Figure 3:**
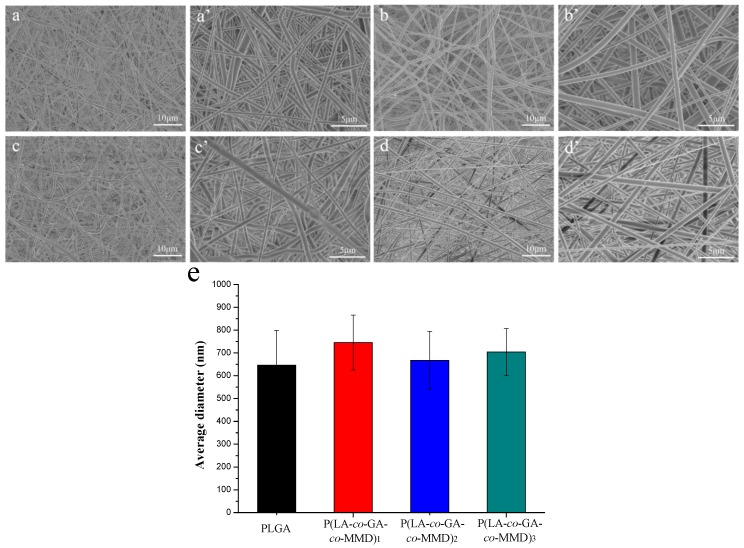
SEM of electrospun nanofibrous scaffolds: (**a** and **a’**) PLGA; (**b** and **b’**) P(LA-*co*-GA-*co*-MMD)_1_; (**c** and **c’**) P(LA-*co*-GA-*co*-MMD)_2_; (**d** and **d’**) P(LA-*co*-GA-*co*-MMD)_3_ and (**e**) average diameters of electrospun scaffolds. (mean ± SD, *n* = 3).

The morphology of the scaffolds plays an important role in vascular tissue engineering. The scaffold fibers should be uniform and smooth. The beaded and non-uniform scaffolds could be avoided by optimizing solvent system, concentration of the spinning solution, applied voltage, flow rate, temperature, target distance, and rotating speed of collector [51]. The morphology of electrospun PLGA and P(LA-*co*-GA-*co*-MMD) scaffolds was characterized by SEM and shown in Figure 3. It revealed that the fibers are randomly oriented and appear to be uniform and smooth. Quantitative analysis indicated fiber diameters of 650 ± 150, 750 ± 120, 670 ± 130, and 700 ± 100 nm for electrospun PLGA, P(LA-*co*-GA-*co*-MMD)_1_, P(LA-*co*-GA-*co*-MMD)_2_, and P(LA-*co*-GA-*co*-MMD)_3_ scaffolds, respectively. There was no significant difference in the fiber diameters among PLGA and those P(LA-*co*-GA-*co*-MMD) scaffolds with different contents of MMD. It might be attributed to the same electrospuning conditions of PLGA and P(LA-*co*-GA-*co*-MMD) scaffolds.

### 3.3. Mechanical Properties of PLGA and P(LA-co-GA-co-MMD) Scaffolds

The mechanical properties of vascular tissue engineering scaffolds are quite important to evaluate whether they are recommended for biomedical application. The mechanical properties of PLGA and P(LA-*co*-GA-*co*-MMD) nanofibrous scaffolds in dry and wet conditions were summarized in Table 2. It’s well known that the tensile strength of natural coronary artery is about 1.4–11.14 MPa, and elongation at break is usually above 40% [52]. Previous studies showed that the tensile strength and elongation at break of PLGA-based nanofibrous scaffolds were about 2–6 MPa and 80%–140% [5,53,54,55,56]. Here, the tensile strength and elongation at break of PLGA and P(LA-*co*-GA-*co*-MMD) nanofibrous scaffolds at dry condition were in the range of 2.63–3.23 MPa and 84%–117%, respectively, which could meet the mechanical requirement for transplantation of vascular scaffolds. There were no significant differences of mechanical properties between electrospun PLGA and P(LA-*co*-GA-*co*-MMD) nanofibrous scaffolds in dry condition. Under the wet condition, the tensile strength and elongation at break of PLGA and P(LA-*co*-GA-*co*-MMD) nanofibrous scaffolds were also in the range of 1.89–2.46 MPa and 114%–559%, respectively. The tensile strength slightly reduced compared with the dry condition for the same nanofibrous scaffold. And the elongation at break of the same nanofibrous scaffold in wet condition was much higher than that in dry condition. Moreover, the elongations at break of scaffolds under wet condition increased significantly with the increasing contents of MMD in copolymers. For P(LA-*co*-GA-*co*-MMD)_3_ nanofibrous scaffold, the elongation at break was 559% ± 82%, significantly higher than the other PLGA-based scaffolds.

**Table 2:** Mechanical properties of PLGA and P(LA-*co*-GA-*co*-MMD) scaffolds under dry and wet conditions.

Sample ID	Tensile Strength (MPa)		Elongation at Break (%)	
	Dry	Wet	Dry	Wet
PLGA	2.89 ± 0.31	2.20 ± 0.12	84 ± 5	114 ± 20
P(LA-*co*-GA-*co*-MMD)_1_	2.63 ± 0.20	2.46 ± 0.27	105 ± 28	242 ± 34
P(LA-*co*-GA-*co*-MMD)_2_	2.66 ± 0.07	1.89 ± 0.46	109 ± 25	363 ± 32
P(LA-*co*-GA-*co*-MMD)_3_	3.23 ± 0.61	2.20 ± 0.68	117 ± 9	559 ± 82

### 3.4. In Vitro Degradation of PLGA and P(LA-co-GA-co-MMD) Scaffolds

**Figure 4:**
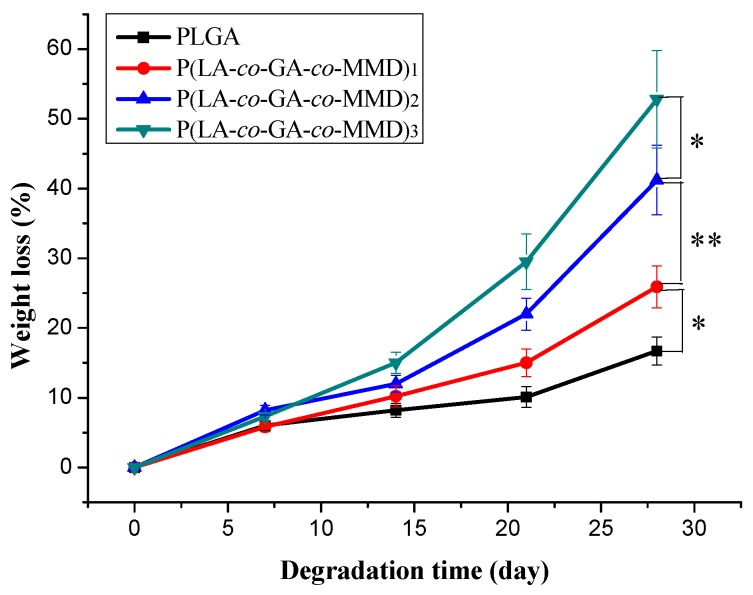
*In vitro* degradation of PLGA and P(LA-*co*-GA-*co*-MMD) nanofibrous scaffolds in PBS solution (pH = 7.4) at 37 °C for 28 days. (mean ± SD, *n* = 3, * *p* ≤ 0.05, ** *p* ≤ 0.01).

Degradation behavior influences directly on the application of the scaffolds. The ideal materials for tissue engineering are desired as temporary scaffolds to support cell growth and disappear with the progress of tissue regeneration. The degradation behavior of electrospun PLGA and P(LA-*co*-GA-*co*-MMD) nanofibrous scaffolds in PBS solution (pH = 7.4) at 37 °C for 28 days was investigated based on their weight loss. As shown in Figure 4, after 14 days of degradation, there was a little weight reduction for all scaffolds. The weight loss was 8.2% ± 1.0%, 10.2% ± 1.6%, 12.3% ± 1.2%, and 14.9% ± 1.5% for PLGA, P(LA-*co*-GA-*co*-MMD)_1_, P(LA-c*o*-GA-*co*-MMD)_2_, and P(LA-*co*-GA-*co*-MMD)_3_ scaffolds, respectively. Then, the weights of all scaffolds were continuously decreased and the P(LA-*co*-GA-*co*-MMD)_3_ nanofibrous scaffold showed the largest weight loss. At 28 days, the weight loss of PLGA, P(LA-*co*-GA-*co*-MMD)_1_, P(LA-c*o*-GA-*co*-MMD)_2_, and P(LA-*co*-GA-*co*-MMD)_3_ were 16.7% ± 2.5%, 25.9% ± 3.4%, 41.2% ± 5.3%, and 52.8% ± 6.8%. Generally, the weight loss of PLGA scaffold was lower than that of P(LA-*co*-GA-*co*-MMD) scaffolds. The degradation behavior was found to depend strongly on the MMD contents in the copolymers and the weight loss of electrospun P(LA-*co*-GA-*co*-MMD) nanofibrous scaffolds increased with the increasing content of MMD in copolymers. It was mainly attributed to the faster hydrolysis of MMD residue, and the MMD residue contains both ester and amide groups in the main chain which ensure high degradability [35,37]. These results suggested that the P(LA-*co*-GA-*co*-MMD) nanofibrous scaffolds had good biodegradable property and the degradability of scaffolds could be controlled by varying the contents of MMD in the copolymers.

### 3.5. Proliferation of HUVECs on PLGA and P(LA-co-GA-co-MMD) Scaffolds

**Figure 5:**
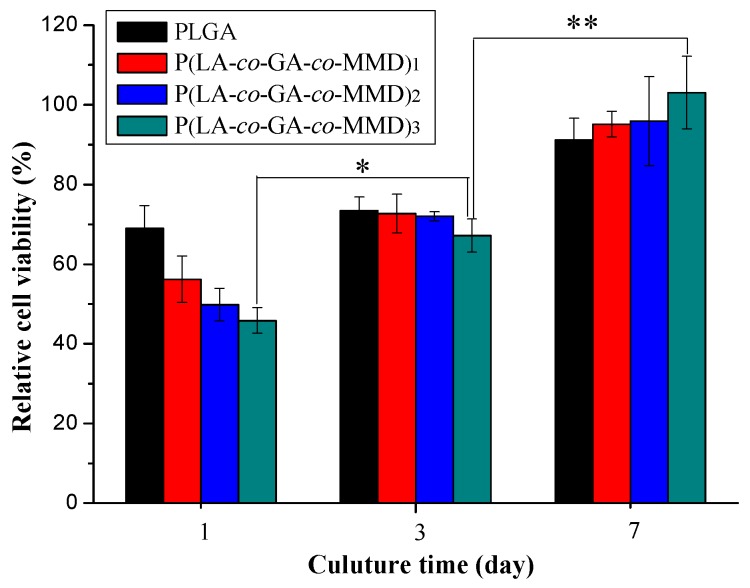
The relative cell viability of HUVECs cultured on PLGA and P(LA-*co*-GA-*co*-MMD) nanofibrous scaffolds. (mean ± SD, *n* = 3, * *p* ≤ 0.05, ** *p* ≤ 0.01).

The proliferation behavior of HUVECs on PLGA and P(LA-*co*-GA-*co*-MMD) nanofibrous scaffolds was determined by MTT assay for one, three, and seven days and the results were shown in Figure 5. The relative cell viability was increasing over time for the same scaffold. At the first day, the relative cell viability of PLGA, P(LA-*co*-GA-*co*-MMD)_1_, P(LA-c*o*-GA-*co*-MMD)_2_, and P(LA-*co*-GA-*co*-MMD)_3_ was 69.0% ± 5.7%, 56.2% ± 5.8%, 49.8% ± 4.1% and 45.8% ± 3.2%, respectively. It demonstrated that the electrospun scaffolds were highly biocompatible and non-cytotoxic, and illustrated that the scaffolds were favorable for cell attachment. However, the relative cell viability on the P(LA-*co*-GA-*co*-MMD) scaffolds was lower than that of PLGA scaffold at the first day. After culturing for seven days, the relative cell viability were 91.2% ± 5.4%, 95.1% ± 3.2%, 95.9% ± 11.2%, and 103.1% ± 9.1% for PLGA, P(LA-*co*-GA-*co*-MMD)_1_, P(LA-c*o*-GA-*co*-MMD)_2_, and P(LA-*co*-GA-*co*-MMD)_3_ nanofibrous scaffolds, respectively. The relative cell viability of HUVECs on P(LA-*co*-GA-*co*-MMD) nanofibrous scaffolds increased with the increasing contents of MMD in copolymers and HUVECs on the P(LA-*co*-GA-*co*-MMD)_3_ scaffolds exhibited the highest relative cell viability. The main reason was the degradation product (l-alanine) of MMD in P(LA-*co*-GA-*co*-MMD) copolymers might promote the proliferation of HUVECs via a signal pathway [43]. These results were in good agreement with our previous studies [42,46].

**Figure 6:**
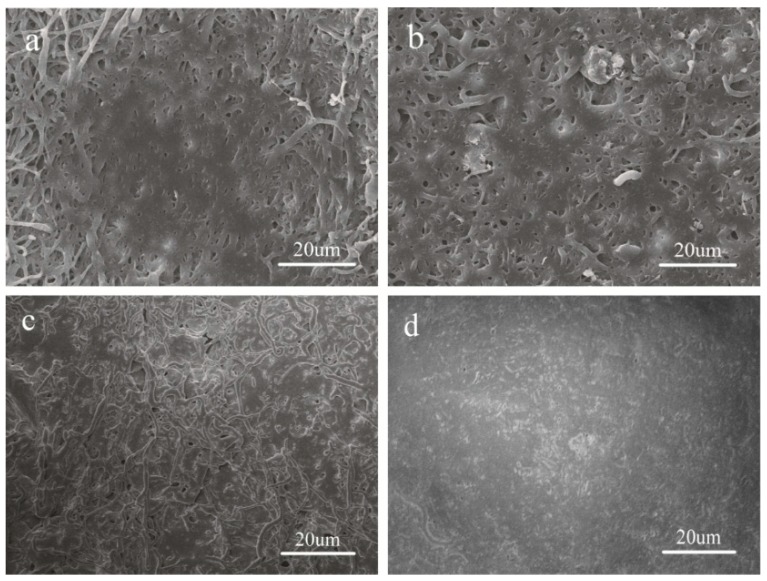
SEM of HUVECs cultured on electrospun nanofibrous scaffolds: (**a**) PLGA; (**b**) P(LA-*co*-GA-*co*-MMD)_1_; (**c**) P(LA-*co*-GA-*co*-MMD)_2_; and (**d**) P(LA-*co*-GA-*co*-MMD)_3_.

To investigate the cell adhesion and proliferation, HUVECs were cultured on electrospun PLGA and P(LA-*co*-GA-*co*-MMD) scaffolds. After culturing for seven days, the cell adhesion behavior was observed by SEM, and the images were presented in Figure 6. It was observed that the HUVECs could adhere and grow well on the surface of all scaffolds; the cells could closely adhere on the fibrous surface and stretch across the randomly interconnected structures. However, considering the areas covered with cells on the scaffolds, the cell numbers of attachment and growth on the P(LA-*co*-GA-*co*-MMD) scaffolds were higher than those of PLGA scaffold, which was consistent with the MTT assay. These results demonstrated that the P(LA-*co*-GA-*co*-MMD) scaffolds could promote the cell adhesion and proliferation.

### 3.6. Tissue Response of Scaffold in Subcutaneous Implantation

**Figure 7:**
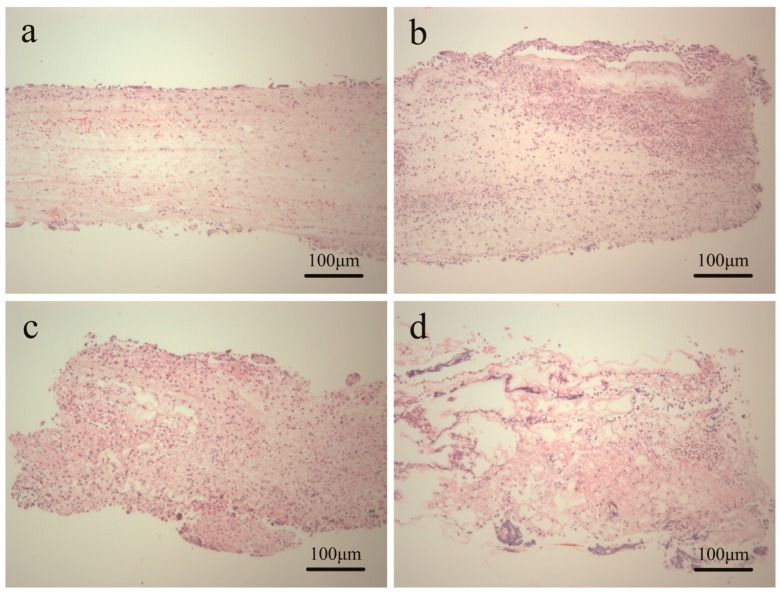
H and E images of subcutaneous implanted scaffolds for seven days: (**a**) PLGA; (**b**) P(LA-*co*-GA-*co*-MMD)_1_; (**c**) P(LA-*co*-GA-*co*-MMD)_2_; and (**d**) P(LA-*co*-GA-*co*-MMD)_3_.

The vascular scaffolds should possess excellent biocompatibility. Biocompatibility indicates the ability of a material to perform with an appropriate tissue response. The tissue responses of scaffolds were evaluated via subcutaneous implantation in Sprague-Dawley rats. Histological analysis was carried out after seven day-implantation in order to evaluate cell infiltration and the representative images were shown in Figure 7. In general, all the explanted scaffolds showed the cell infiltration. For PLGA scaffold, the cell attachment and infiltration mainly occurred on the surface of the scaffold and only a few cells could infiltrate deeply into the internal structure. The case of P(LA-*co*-GA-*co*-MMD) scaffolds was completely different, where most of the cells had infiltrated deeply into the internal structure. The possible reason was that P(LA-*co*-GA-*co*-MMD) scaffolds could undergo relatively fast degradation after subcutaneous implantation. As a result, some fibers were broken and the pore size of the scaffolds became large, which was favorable for the cells easily to infiltrate deeply into the internal structure.

In order to overcome the thrombogenicity and compliance mismatch in small diameter vessel reconstruction, researchers have focused on tissue engineering and biodegradable scaffolds to develop “living” vascular grafts. The ideal vascular scaffolds should provide the initial strength, and then gradually degrade and cells grow to create a graft composed solely of host tissue. We have developed a series of biodegradable scaffolds based on P(LA-*co*-GA-*co*-MMD) copolymers. It showed excellent degradation behavior compared with PLGA scaffold, and the degradability of the P(LA-*co*-GA-*co*-MMD) scaffolds could be controlled by varying the contents of MMD in the copolymers. Furthermore, the scaffolds could provide a suitable environment for ECs to adhere, grow and proliferate.

## 4. Conclusions

In this study, P(LA-*co*-GA-*co*-MMD) copolymers were synthesized by ROP of LA, GA, and MMD using Sn(Oct)_2_ as catalyst. Then, biodegradable P(LA-*co*-GA-*co*-MMD) scaffolds were prepared by electrospinning technology. The P(LA-*co*-GA-*co*-MMD) scaffolds showed good mechanical properties and the weight loss is higher than PLGA scaffolds, which indicates the good biodegradable property of the scaffold. In addition, the HUVECs could attach and proliferate well on the P(LA-*co*-GA-*co*-MMD) scaffolds to form a cell monolayer fully covering on scaffold surface. Furthermore, cells could infiltrate well into the P(LA-*co*-GA-*co*-MMD) scaffolds after subcutaneous implantation. These results indicated that the P(LA-*co*-GA-*co*-MMD) nanofibrous scaffolds could be potential candidates for vascular tissue engineering.
